# Evaluation of Hepatoprotectants in the Management of Subclinical Gallbladder Mucocele in Dogs

**DOI:** 10.3390/ani15203002

**Published:** 2025-10-16

**Authors:** Jiyoon Lee, Jiyoung Park, Sang-Joon Lee, Changbaig Hyun

**Affiliations:** Animal Heart Institute, VIP Animal Medical Center, Chungdam-dong, Kangnam-Gu, Seoul 06078, Republic of Korea; hellojyl@gmail.com (J.L.); pkjy0807@gmail.com (J.P.); sjoon516@gmail.com (S.-J.L.)

**Keywords:** gallbladder mucocele, hepatoprotectants, liver function markers, S-adenosylmethionine (SAMe), Ursodeoxycholic acid (UDCA)

## Abstract

**Simple Summary:**

This study investigated the effects of different hepatoprotectants on subclinical gallbladder mucocele (GBM) in dogs. Sixty dogs were divided into three groups: UDCA alone, SAMe with silymarin, or a combination of UDCA, SAMe, and silymarin, and monitored for one year. The combination therapy (Group 3) produced the best outcomes, with significant reductions in liver enzyme levels (GGT, ALP, ALT, AST), a marked decrease in gallbladder sludge, and improved liver echogenicity on ultrasound. UDCA alone led to mild improvements, while SAMe with silymarin had limited effects, mostly on ALT and AST but not on gallbladder changes. Overall, the findings suggest that combined therapy with UDCA, SAMe, and silymarin is more effective than monotherapy for managing subclinical GBM in dogs. The study supports medical management as a potential option but highlights the need for further research into long-term outcomes and mechanisms.

**Abstract:**

Gallbladder mucocele (GBM) in dogs is a condition characterized by the excessive accumulation of mucin within the gallbladder, potentially leading to bile duct obstruction and serious complications. While cholecystectomy remains the treatment of choice for symptomatic cases, medical management is often considered in dogs with subclinical GBM. This study evaluated the effects of different hepatoprotectants on disease progression in subclinical GBM. Sixty dogs diagnosed with GBM were randomly assigned to one of the three treatment groups: Group 1 (Ursodeoxycholic acid (UDCA) alone), Group 2 (S-adenosylmethionine (SAMe) and silymarin), and Group 3 (UDCA, SAMe, and silymarin). Hepatic biochemical markers (GGT, ALP, ALT, AST, bilirubin, cholesterol) and ultrasound parameters (gallbladder sludge percentage, liver echogenicity) were assessed at baseline, day 30, day 60, day 180, and day 365. Group 3 exhibited the most significant improvement, with substantial reductions in GGT, ALP, ALT, and AST levels (*p* < 0.05). Group 3 also demonstrated a significant decrease in gallbladder sludge percentage and improved liver echogenicity (*p* < 0.05). Group 1 showed mild improvement, whereas Group 2 had minimal impact on markers of cholestasis or gallbladder health. These findings suggest that a combination therapy of UDCA, SAMe, and silymarin may offer the most effective medical approach for managing subclinical GBM in dogs.

## 1. Introduction

A gallbladder mucocele (GBM) in dogs is a condition characterized by the accumulation of thick, mucin-rich bile within the gallbladder, leading to its distension [1,2]. This can result in bile duct obstruction and, if untreated, may cause gallbladder rupture, bile peritonitis, and potentially life-threatening complications. Gallbladder mucocele (GBM) has increasingly been recognized in small animal practice, with prevalence estimates ranging from 1.5% to 2.5% among dogs undergoing abdominal ultrasonography [3]. Certain breeds such as Shetland Sheepdogs, Cocker Spaniels, and Pomeranians are disproportionately affected, suggesting a genetic or metabolic predisposition [4,5]. Despite this, the natural history of subclinical GBM remains unclear, as some cases remain stable for years while others progress rapidly to clinical disease or gallbladder rupture [6]. These epidemiological uncertainties justify prospective observational studies to better define disease trajectories and treatment responses. The exact cause of GBM formation remains unclear, but several factors have been associated with its development, such as breed predisposition, age and endocrinopathies, hyperlipidemia, and gallbladder dysmotility [3,7]. Dogs with GBM may present with a range of clinical signs, from asymptomatic cases to severe illness [2]. Diagnosis involves a combination of blood tests (e.g., elevated liver enzymes), increased bilirubin levels, and hypercholesterolemia) and abdominal imaging (e.g., “kiwi-like” appearance gallbladder). Cholecystectomy is the treatment of choice, especially for dogs with severe clinical signs or evidence of gallbladder rupture [2]. In asymptomatic or early-stage cases, medical management may be considered. This includes administering choleretic agents like ursodiol to promote bile flow, antioxidants such as S-adenosylmethionine (SAMe), antibiotics if bacterial infection is suspected, and dietary modifications to a low-fat diet. The prognosis varies based on the stage at diagnosis and the treatment approach.

Ursodeoxycholic acid (UDCA), also known as ursodiol, is a secondary bile acid widely used in veterinary medicine to treat hepatobiliary disorders, including GBMs in dogs [8]. UDCA promotes bile flow and reduces the cholesterol content of bile, making it particularly useful in conditions associated with bile stasis. In dogs without clinical signs or evidence of gallbladder rupture or biliary obstruction, medical management with UDCA can be considered. Its mechanism of action includes inducing hydrocholeresis, which enhances bile secretion and potentially prevents further accumulation of mucus within the gallbladder [9].

While UDCA can be beneficial in certain cases, its ability to resolve established GBMs is limited. S-adenosylmethionine (SAMe) is a naturally occurring compound involved in essential metabolic pathways, including methylation and transsulfuration. It serves as a precursor to glutathione, a key intracellular antioxidant. In veterinary medicine, SAMe is commonly administered as a hepatoprotectant to support liver function and protect hepatocytes from oxidative damage [8]. However, its specific efficacy in treating or preventing GBMs remains uncertain [9]. SAMe’s is predicted to be of help in resolution of GBM due to it extensively reported antioxidant properties [10,11]. SAMe is often included in multimodal medical protocols for dogs with subclinical GBM, despite the lack of evidence supporting its standalone effectiveness. Silymarin, an extract from the milk thistle plant (*Silybum marianum*), is renowned for its hepatoprotective properties and is commonly used to support liver function in both humans and animals. In veterinary medicine, silymarin is often administered to dogs to safeguard liver health, particularly in conditions that may compromise hepatic function [8]. While silymarin’s antioxidant and anti-inflammatory effects are beneficial for liver health, its direct impact on gallbladder mucoceles (GBMs) in dogs is not well-established.

The treatment of subclinical GBM in dogs is unclear and is not well studied to date. However, treatment with UDCA, SAMe, a low-fat diet, and screening for endocrinopathies is generally recommended in dogs with subclinical GBM, although there is also no strong evidence that medical therapy alters disease progression [2,8]. Therefore, this study was designed for evaluating the effect of hepatoprotectants on disease progression in dogs with subclinical GBM.

## 2. Materials and Methods

This was designed as a prospective observational cohort study. Dogs were consecutively diagnosed with subclinical GBM at a referral hospital between 2021 and 2023. Although case inclusion was determined at the time of diagnosis (retrospective identification), subsequent monitoring, treatment allocation, and follow-up evaluations were performed prospectively at predetermined time points (day 0, 30, 60, 180, and 365). Sixty dogs diagnosed with GBM from 2021 to 2023 at VIP Animal Medical Center, Seoul were enrolled in this study. Dogs enrolled in this study did not receive any concurrent hepatoprotective agents, antibiotics, or dietary modifications (e.g., prescription low-fat diets) outside the assigned treatment regimens. Compliance with follow-up was high, with ≥18 of 20 dogs in each group attending at each timepoint, and data were analyzed on an available-case basis. No dogs were withdrawn from the study, and no treatment-related complications were recorded during the 12-month monitoring period. Diagnostic criteria similar to those previously described were applied [12]. Specifically, a diagnosis of GBM was confirmed by abdominal ultrasonography based on the presence of non-gravity-dependent, immobile, stellate, or “kiwi-like” hyperechoic material completely filling or occupying the majority of the gallbladder lumen [12]. For the purpose of this study, “subclinical GBM” was operationally defined as the presence of ultrasonographic findings consistent with gallbladder mucocele (i.e., immobile, gravity-independent sludge and/or echogenic bile not altering with positional changes) in dogs without clinical signs (e.g., anorexia, vomiting, jaundice, abdominal pain) and without evidence of biliary obstruction or gallbladder rupture. Histological confirmation was not obtained due to the non-invasive nature of the study design. Dogs were included only when these criteria were fulfilled in conjunction with biochemical evidence of normal or mildly elevated liver enzyme activities. Owners were instructed to maintain the dog’s pre-study diet unless concurrent conditions required a specific prescription diet. No mandatory low-fat diet or standardized dietary protocol was implemented across the three study groups during the one-year study period. Any dog having other systemic diseases including endocrinopathies were excluded from this study. Sixty dogs were randomly divided into 3 different groups (*n* = 20, each) and then medicated with 3 different combinations of hepatoprotectants for 1 year: Group 1 (UDCA 10–15 mg/kg, q24h; UDCA tablet, Daewoong Pet, Seoul, Republic of Korea), Group 2 (SAMe 20 mg/kg, q24h, silymarin 5–10 mg, q24h; Samylin, Vetplus, Lancashire, UK), and Group 3 (UDCA 10–15 mg/kg, q24h, SAMe 20 mg/kg, q24h, silymarin 5–10 mg, q24h; Usasil, Careside, Sungnam, Republic of Korea). The commercial formulations used in this study contained the active ingredients as indicated (UDCA, SAMe, silymarin). According to information available from the manufacturers, additional components were limited to excipients or flavoring agents and were not expected to have pharmacological effects on liver function. Nevertheless, it cannot be excluded that the superior results observed in Group 3 may be attributable in part to unique formulation characteristics or bioavailability of the specific commercial product (Usasil), rather than a synergistic effect of the three active compounds alone. Doses within the specified ranges were selected for each dog based on body weight and the available tablet/capsule sizes to allow practical administration as close as possible to the target dosage. Thus, minor variability in dosage within a group reflected formulation constraints rather than intentional dose titration. Blood biochemistry for hepatic function (i.e., alkaline phosphatase [ALP], alanine transaminase [ALT], aspartate aminotransferase [AST], gamma-glutamyl transferase [GGT], bilirubin and cholesterol; Catalyst One Veterinary Blood Chemistry Analyzer, Idexx, Westbrook, ME, USA) and abdominal ultrasonography (AUS) for gall bladder findings were serially evaluated at day 0 (baseline), day 30, day 60, day 180 and day 365. Ultrasound examinations were performed in all cases by board-certified radiologists. Ultrasonography was performed in all cases using a Toshiba Aplio i700 (Toshiba medical systems corporation, Otawara City, Japan) system. The maximal proportion (%) of gallbladder mucocele was evaluated with ultrasonography if a gallbladder contained gravity-independent and immobile material that did not change with position of the dog. The change of hepatic parenchyma on the ultrasound exam were evaluated and graded by the following score system: score 1 (No change in liver size but having 1–2 focal lesions with altered echogenicity), score 2 (No change in liver size but having multiple lesions with altered echogenicity), score 3 (Diffuse increase in size with altered echogenicity), and score 4 (Decreased liver size with altered echogenicity and irregular contour). The inter-observer reliability of the ultrasound measurement/interpretation was evaluated using a cross-sectional design. A total of 60 dogs/ultrasound images were independently assessed by 2 qualified observers. The observers were blinded to the clinical data and the results of the other raters.

Categorical data were anonymized and recorded, with each category presented descriptively. Continuous data (including age in years, blood biochemistry results) were recorded and values for mean and standard deviations were calculated for each variable. Data were assessed for normality using the Kolmogorov–Smirnov test and for homogeneity of variance using Levene’s test. Repeated measures ANOVA was applied to evaluate changes over time and between groups. When the assumption of sphericity was violated (Mauchly’s test), Greenhouse–Geisser correction was applied. Effect sizes (partial η^2^) and 95% confidence intervals were calculated to estimate the magnitude and precision of treatment effects. When ANOVA results were significant, post hoc pairwise comparisons were conducted with Bonferroni correction to control for multiple testing. Exact *p*-values are reported throughout. All statistical analyses were performed using MedCalc^®^ Statistical Software, version 23.1.7 (MedCalc Software Ltd., Ostend, Belgium). Results were considered significant if *p* < 0.05.

## 3. Results

There were no significant differences in age, gender, breed composition, body weight and body condition score among groups (Table 1, *p* > 0.05). All dogs enrolled in this study survived at the end of the study. The three most common breeds (Maltese, Pomeranian, and Poodle) accounted for over 65% of the total population.

**Figure 1:**
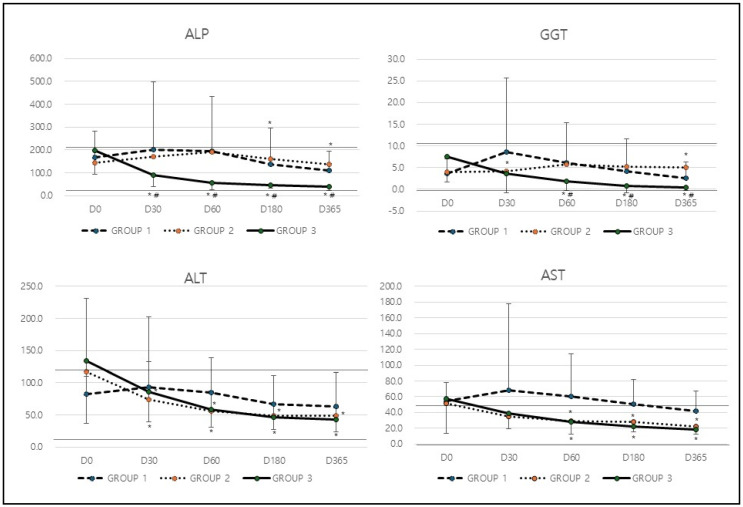
Changes in hepatic biochemical markers (ALP, GGT, ALT, AST) after treatment. Dotted lines indicate upper and lower reference range. * *p* < 0.05 compared with baseline (D0) value, ^#^ *p* < 0.05 compared among groups. GGT (Gamma-Glutamyl Transferase), ALP (Alkaline Phosphatase), ALT (Alanine Aminotransferase), AST (Aspartate Aminotransferase).

In analysis of hepatic biochemical markers (Table 2), the levels of GGT in Group 1 increased from 3.6 at baseline to 8.6 at D30 but subsequently decreased to 2.6 by D365 (* *p* = 0.032). In contrast, Group 2 exhibited relatively stable GGT levels, starting at 3.9 and slightly increasing to 5.1 at D365. Group 3, however, showed a consistent and substantial reduction in GGT levels, dropping from 7.5 at baseline to 0.4 at D365 (* *p* = 0.0001 compared to baseline, ^#^ *p* = 0.001 compared among groups; Table 2 & Figure 1). The most significant decrease in GGT levels was observed in Group 3, indicating that the treatment applied to this group was the most effective in improving cholestasis. Similarly to GGT, Group 1 experienced an increase in ALP levels from 169.2 at baseline to 202.4 at D30, followed by a decline to 109.2 at D365 (* *p* = 0.015). Group 2 showed only minor fluctuations, with ALP levels rising from 144.9 to 190.3 by D60 before slightly decreasing to 139.0 by the end of the study period. In contrast, Group 3 demonstrated a steady and significant reduction, with ALP levels dropping from 196.5 at baseline to 38.9 at D365 (* *p* = 0.0001, ^#^ *p* = 0.0021; Table 2 & Figure 1). The greatest reduction in ALP levels occurred in Group 3, suggesting that this treatment had the most pronounced positive effect on cholestasis.

In the analysis of hepatic leakage markers such as ALT and AST, ALT levels in Group 1 initially increased from 82.7 to 92.8 at D30 but then gradually declined to 63.1 by D365. Group 2 displayed a notable reduction from 117.3 at baseline to 49.4 by D365 (* *p* = 0.023). Similarly, Group 3 showed a significant decrease, with ALT levels dropping from 134.1 to 42.4 over the study period (* *p* = 0.0001; Table 2 & Figure 1). Both Group 2 and Group 3 experienced significant reductions in ALT levels, indicating an improvement in liver health and function. Similarly to ALT, Group 1 exhibited only a minor decrease in AST levels, which fell from 54.1 to 41.5 by D365. In contrast, Group 2 demonstrated a significant reduction from 52.0 to 22.1 (* *p* = 0.032), and Group 3 displayed an even greater decrease, with AST levels declining from 57.2 to 18.5 by the end of the study period (* *p* = 0.0001; Table 2 & Figure 1). The most substantial improvement in AST levels was observed in Group 3, while Group 2 also experienced a significant reduction. This suggests that the treatments in these groups were effective in reducing liver inflammation and improving overall liver function.

**Figure 2:**
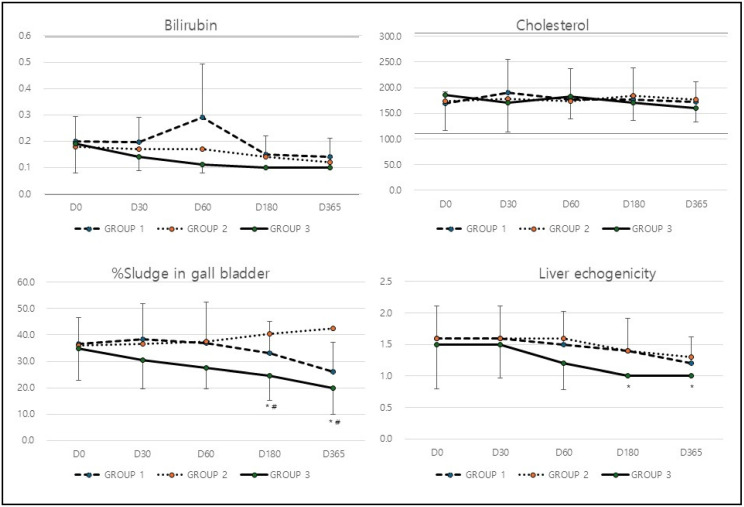
Changes in hepatic biochemical (bilirubin & cholesterol) and ultrasound markers (% Sludge in GB & liver echogenicity) after treatment. Dotted lines indicate upper and lower reference range. * *p* < 0.05 compared with baseline (D0) value, ^#^ *p* < 0.05 compared among groups.

Unlike other hepatic markers, the bilirubin and cholesterol levels remained largely unchanged across all three groups, with only minor fluctuations that were not statistically significant (*p* > 0.05, Table 2 and Figure 2). The treatment did not appear to have any significant impact on bilirubin or cholesterol levels.

In analysis of hepatic ultrasound markers (Table 3), the percentage of sludge in the gallbladder increased slightly from 36.5% at baseline to 38.5% at D30 but then declined to 26.0% by D365 (* *p* = 0.043) in Group 1. Group 2, on the other hand, showed a continuous increase in gallbladder sludge, rising from 36.0% to 42.5% over the study period. In contrast, Group 3 experienced a significant reduction, with sludge levels decreasing from 35.0% to 20.0% by the end of the study (* *p* = 0.0001, ^#^ *p* = 0.0001; Table 3 and Figure 2). The most substantial reduction in gallbladder sludge was seen in Group 3, indicating that the treatment in this group effectively enhanced liver and gallbladder function. Similarly to the effect on gall bladder, liver echogenicity remained unchanged at 1.6 until D60, then improved slightly to 1.2 by D365 in Group 1. Group 2 followed a similar pattern, with echogenicity improving from 1.6 to 1.3 by the end of the study. Group 3, however, displayed the most significant improvement, with echogenicity declining from 1.5 at baseline to 1.0 at D365 (* *p* = 0.012; Table 3 and Figure 2). The significant improvement in liver echogenicity observed in Group 3 suggests a marked improvement in liver tissue characteristics and a potential reduction in fatty liver infiltration.

The data indicates that the treatment applied to Group 3 was the most effective in improving both hepatic biochemical markers and ultrasound markers. This group experienced significant reductions in liver enzyme levels, a decrease in gallbladder sludge percentage, and an improvement in liver echogenicity, all of which suggest enhanced liver function and overall hepatic health. Although the treatment used in Group 2 showed some effectiveness in reducing ALT and AST levels, it did not have noticeable beneficial effect on the gall bladder and cholestasis. The treatment used in Group 1 exhibited only mild improvements across most markers, indicating that its treatment was the least effective among the three groups.

## 4. Discussion

This study demonstrated significant improvements in hepatic biochemical markers and ultrasonographic parameters following treatment, with Group 3 (UDCA, SAMe, and silymarin) showing the most substantial benefits. The results indicate reductions in liver enzyme levels (GGT, ALP, ALT, and AST), a decrease in gallbladder sludge, and an improvement in liver echogenicity. To assess the implications of these findings, it is essential to compare them with previous studies that have explored similar treatments and their impact on liver health.

Elevated GGT levels are commonly associated with liver damage, oxidative stress, and biliary dysfunction. Previous studies conducted in humans have shown that antioxidant-based treatments and hepatoprotective agents can transiently reduce GGT levels [13]. For instance, a randomized clinical trial in patients with chronic hepatitis C reported an approximate 50% reduction in GGT levels after six months of silymarin supplementation; however, this effect was not sustained at 12 months [14]. Furthermore, a subsequent systematic review and meta-analysis concluded that pooled data did not demonstrate significant overall changes in GGT levels with silymarin therapy [15]. This discrepancy underscores the variability of reported outcomes and suggests that the benefits of silymarin are not consistent across populations or disease states. In our study, Group 3 demonstrated a steady decrease in GGT, reaching a near-normal level by day 365, suggesting that the combined regimen may share antioxidant or hepatoprotective properties, although the contribution of each component remains

Interestingly, Group 1 (UDCA alone) exhibited a transient increase in both GGT and ALP levels at day 30 before subsequently declining by day 365. Similar transient elevations in liver enzymes following the initiation of UDCA therapy have been reported in both veterinary and human studies, and are thought to reflect adaptive changes in bile acid metabolism rather than true worsening of disease. Importantly, by the end of the study period, Group 1 demonstrated a significant reduction in ALP levels. In contrast, Group 2 (SAMe with silymarin, without UDCA) exhibited no meaningful change in ALP. This observation further supports the role of UDCA in mitigating cholestatic injury, and suggests that the improvement in cholestatic markers observed in Group 3 may be primarily attributable to UDCA.

ALP is a marker of cholestatic liver injury. In a human cohort of patients who developed gallstones after laparoscopic sleeve gastrectomy, UDCA treatment was associated with a significant reduction in ALP levels [16]. Although the patient population and disease condition differ from canine GBM, this finding is consistent with our results and supports the hypothesis that UDCA can improve biochemical markers of cholestasis in settings of biliary stasis.

Elevated ALT and AST are hallmarks of hepatocellular injury. Previous studies [17] have shown that reductions in these enzymes correlate with hepatoprotective interventions, such as polyphenol-based supplementation and lifestyle modifications. Our study reinforces this observation, particularly in Group 3, where ALT and AST levels significantly decreased over time. This suggests that the regimen in this group may have incorporated anti-inflammatory or hepatoprotective mechanisms similar to those reported in earlier research. SAMe, in particular, is widely recognized as a precursor of glutathione and a cellular antioxidant. However, clinical evidence for its efficacy in canine GBM remains limited. The reductions in ALT and AST observed in Group 2 (SAMe with silymarin) may reflect SAMe’s hepatocellular protective effects, although the absence of changes in cholestatic markers or gallbladder sludge indicates that its benefits may be restricted to hepatocyte integrity rather than biliary function.

Gallbladder sludge is often associated with biliary stasis and liver dysfunction. An experimental study in mice demonstrated that UDCA treatment led to a significant reduction in gallbladder sludge over a one-year period [18]. Similarly, in our study, Group 3 exhibited a significant reduction in sludge percentage, dropping from 35.0% to 20.0% by day 365, indicating improved biliary function. In contrast, Group 2, the only group that did not receive UDCA, showed an increase in sludge percentage, suggesting that UDCA was a critical factor in improving biliary stasis.

Increased liver echogenicity is often associated with fatty liver disease. One study demonstrated that weight loss, dietary modifications, and antioxidant therapy can lead to significant reductions in liver echogenicity [19]. In our study, Group 3 showed the most notable improvement in liver echogenicity, suggesting that the treatment employed in this group may have contributed to reduced hepatic steatosis.

When interpreting these findings, it is important to clarify the context of prior literature. Several studies cited here were performed in humans (e.g., hepatitis C patients, bariatric surgery cohorts) or in rodent models of fatty liver disease. While informative, these findings should not be directly extrapolated to dogs with GBM. More relevant canine studies include those by Choi et al. [3], which reported ultrasonographic and clinical correlations in dogs with GBM, and Malek et al. [7], which described outcomes of surgical management. Our results extend this body of canine-specific literature by providing prospective, longitudinal data on medical management of subclinical GBM.

Based on comparisons with previous studies, the observed improvements in Group 3 could be attributed to the following possible mechanisms: (1) Hepatoprotective and anti-inflammatory effects: Similar to treatments involving silymarin, polyphenols, or N-acetylcysteine (NAC), the therapy applied to Group 3 may have reduced oxidative stress and inflammation, leading to lower ALT, AST, and GGT levels. (2) Cholestasis prevention: The significant drop in ALP and gallbladder sludge percentage suggests that Group 3’s treatment might have included bile acid-modifying effects (such as those of UDCA) or substances promoting bile flow. (3) Reduction in hepatic fat content: The improvement in liver echogenicity implies that the treatment may have influenced hepatic lipid metabolism, akin to interventions seen in studies involving weight loss, metformin, or omega-3 fatty acids [19].

Overall, the results of this study should be interpreted cautiously. While Group 3 was associated with the most consistent improvements in biochemical and ultrasonographic markers, these findings reflect associations rather than proof of therapeutic superiority. Further randomized, controlled studies in dogs are warranted to confirm whether these improvements translate into clinically meaningful outcomes such as survival or prevention of GBM progression.

The present study should be interpreted within the broader epidemiological context. While our findings suggest that hepatoprotective regimens may influence biochemical and imaging markers in subclinical GBM, the absence of survival or progression data limits conclusions about long-term outcomes. Larger epidemiological studies are required to determine incidence, risk factors for progression, and the external validity of our results.

Although this study presents compelling evidence, there are some limitations: (1) The ultrasonographic evaluations were performed by a single board-certified radiologist. While this ensured internal consistency, inter-observer reproducibility of imaging-based classifications (such as sludge percentage and hepatic echogenicity) was not assessed. As a result, potential misclassification bias due to subjective interpretation cannot be completely excluded. Future studies should incorporate independent assessments by multiple observers and utilize agreement statistics (e.g., Cohen’s kappa, intraclass correlation coefficients) to improve diagnostic consistency and reliability. (2) Although multiple statistical safeguards were applied, including assumption testing and Bonferroni adjustment, the relatively small sample size may limit the generalizability of effect size estimates. Future studies with larger cohorts should confirm these findings with robust multivariable analyses. (3) The study did not include adjusted statistical models, effect size estimates, or survival analyses; therefore, conclusions regarding clinical efficacy or disease resolution cannot be definitively drawn. The observed improvements should be regarded as associations with treatment rather than proof of therapeutic superiority. (4) While follow-up and monitoring were prospective, case enrollment relied on dogs diagnosed at a referral hospital during routine practice. Therefore, elements of retrospective case selection cannot be excluded, which may introduce potential selection bias. Future investigations with strictly prospective recruitment and randomized allocation would provide stronger evidence. (5) Although the active ingredients and dosages were disclosed in the Materials and Methods, the use of proprietary commercial combination products for Group 2 (Samyline) and Group 3 (Usasil) means that direct comparisons with studies using individual, isolated agents remain speculative, as we cannot rule out synergistic effects or the influence of excipients. (6) The lack of a standardized diet across all groups is a limitation, as dietary modifications (such as a low-fat diet) could independently influence liver echogenicity. Lifestyle and genetic factors may also have influenced the outcomes. (7) Histological confirmation of GBM was not available, as diagnosis relied exclusively on ultrasonographic findings. While this is consistent with current clinical practice for subclinical cases, it may not fully capture the histopathological spectrum of disease. Future research should focus on identifying the exact biochemical and pharmacological mechanisms underlying the observed improvements. Controlled trials comparing different treatment regimens could provide more conclusive evidence regarding the efficacy of various interventions in liver disease management.

## 5. Conclusions

In conclusion, this study demonstrated that hepatoprotective treatments were associated with improvements in liver function markers and ultrasonographic parameters in dogs with subclinical GBM. Among the regimens evaluated, the combination of UDCA, SAMe, and silymarin was associated with the most notable improvements in biochemical and imaging markers. However, given the observational nature of this study and the absence of adjusted effect size estimates or survival outcomes, these findings should be interpreted cautiously. Future randomized, controlled, and long-term studies are needed to determine whether such improvements translate into clinical efficacy or disease resolution.

## Figures and Tables

**Table 1:** Demographic distribution of this study population.

	Group 1	Group 2	Group 3
Age (y)	11.2 ± 3.1	10.3 ± 3.5	11.7 ± 3.1
BW (kg)	3.54 (2.92–5.50)	3.67 (2.88–6.18)	3.89 (3.12–6.52)
BCS	5 (4–5)	5 (4–6)	5 (4–6)
Breed *	M (5), P (5), SH (3), PO (4), Mx (2), B (1)	M (6), P (3), SH (4), PO (3), YT (2), Mx (2)	M (5), PO (5), SH (2), PO (3), Mx (2), CS (2), YT (1)
Sex ^#^	SF (10), CM (10)	F (1), SF (9), CM (10)	SF (10), M (1), CM (9)

BCS (Body condition score). * Breed: M (Maltese), P (toy Poodle), SH (Shih Tzu), PO (Pomeranian), Mx (mixed), B (Bishon Frise), YT (Yorkshire terrier), CS (Cocker Spaniel). ^#^ Sex: SF (Spayed female), F (intact female), M (intact male), CM (Castrated male).

**Table 2:** Changes in hepatic biochemical markers after treatment.

		D0	D30	D60	D180	D365
	GROUP 1	3.6	8.6	6.1	4.1	2.6 *
	SD (±)	3.4	17.1	9.2	7.5	3.7
	95% CI	2.54–4.65	3.30–13.89	3.24–8.95	1.77–6.42	1.45–3.74
GGT	GROUP 2	3.9	4.2	5.7	5.3	5.1
	SD (±)	3.1	6.6	3.2	4.2	3.7
	95% CI	2.93–4.86	2.15–6.24	4.70–6.69	3.99–6.60	3.95–6.24
	GROUP 3	7.5	3.6 *	1.9 *^,#^	0.8 *^,#^	0.4 *^,#^
	SD (±)	5.8	4.4	2.2	1.5	0.7
	95% CI	5.70–9.29	2.23–4.96	1.21–2.58	0.33–1.26	0.18–0.61
	GROUP 1	169.2	202.4	194.1	135.9 *	109.2 *
	SD (±)	113.0	294.2	240.1	159.0	84.5
	95% CI	128.7–209.6	97.1–307.6	108.3–279.9	79.0–192.7	78.9–139.4
ALP	GROUP 2	144.9	170.4	190.3	161.0	139.0
	SD (±)	105.9	144.9	170.2	89.7	68.8
	95% CI	107.0–182.7	118.5–222.2	129.4–251.1	128.9–193.1	114.3–163.6
	GROUP 3	196.5	91.0 *^,#^	56.7 *^,#^	46.2 *^,#^	38.9 *^,#^
	SD (±)	101.9	49.9	31.1	13.4	15.5
	95% CI	160.0–232.9	73.1–108.8	45.5–67.8	41.4–50.9	33.3–44.4
	GROUP 1	82.7	92.8	85.3	66.6	63.1
	SD (±)	27.4	109.5	53.8	44.9	53.4
	95% CI	72.9–92.5	53.6–131.9	66.0–104.5	50.5–82.6	44.0–82.2
ALT	GROUP 2	117.3	74.5 *	55.8 *	49.3 *	49.4 *
	SD (±)	52.5	26.1	20.6	20.3	15.2
	95% CI	98.5–136.0	65.1–83.8	48.4–63.1	42.0–56.5	43.9–54.8
	GROUP 3	134.1	86.0 *	59.0 *	46.7 *	42.4 *
	SD (±)	97.6	47.2	27.9	18.9	18.4
	95% CI	99.1–169.0	69.1–102.8	49.0–68.9	39.9–53.4	35.8–48.9
	GROUP 1	54.1	68.2	60.3	50.3	41.5
	SD (±)	24.2	109.3	53.8	32.0	26.1
	95% CI	45.4–62.7	29.0–107.3	41.0–79.5	38.8–61.7	32.1–50.8
AST	GROUP 2	52.0	34.8	28.8 *	28.2 *	22.1 *
	SD (±)	22.4	18.2	15.7	15.6	12.4
	95% CI	43.9–60.0	28.2–41.3	23.1–34.4	22.6–33.7	17.6–26.5
	GROUP 3	57.2	39.3	28.2 *	22.6 *	18.5 *
	SD (±)	43.8	20.2	15.5	7.5	6.0
	95% CI	41.5–72.8	32.0–46.5	22.6–33.7	19.9–25.2	16.3–20.6
	GROUP 1	0.2	0.2	0.3	0.2	0.1
	SD (±)	0.1	0.1	0.2	0.1	0.1
	95% CI	0.16–0.23	0.16–0.23	0.22–0.37	0.16–0.23	0.06–0.13
Bilirubin	GROUP 2	0.2	0.2	0.2	0.1	0.1
	SD (±)	0.1	0.1	0.1	0.1	0.0
	95% CI	0.16–0.23	0.16–0.23	0.16–0.23	0.06–0.13	0.1–0.1
	GROUP 3	0.2	0.1	0.1	0.1	0.1
	SD (±)	0.1	0.1	0.0	0.0	0.0
	95% CI	0.16–0.23	0.06–0.13	0.1–0.1	0.1–0.1	0.1–0.1
	GROUP 1	169.7	190.3	176.6	177.5	171.7
	SD (±)	22.7	64.4	61.0	61.5	40.6
	95% CI	161.5–177.8	167.2–213.3	154.7–198.4	155.4–199.5	157.1–186.2
Cholesterol	GROUP 2	174.1	178.8	173.9	184.1	177.5
	SD (±)	54.3	71.8	60.5	39.4	26.9
	95% CI	154.6–193.5	153.1–204.4	152.2–195.5	170.0–198.1	167.8–187.1
	GROUP 3	185.8	171.5	183.6	171.2	161.0
	SD (±)	69.0	58.3	44.1	35.0	28.3
	95% CI	161.1–210.5	150.6–192.3	167.8–183.7	158.6–183.7	150.8–171.1

GGT (Gamma-Glutamyl Transferase), ALP (Alkaline Phosphatase), ALT (Alanine Aminotransferase), AST (Aspartate Aminotransferase), CI (confidence interval). Exact *p*-values are reported in the text. * *p* < 0.05 compared with baseline (D0) value, ^#^ *p* < 0.05 compared among groups.

**Table 3:** Changes in hepatic ultrasound markers after treatment.

		D0	D30	D60	D180	D365
	GROUP 1	36.5	38.5	37.0	33.0	26.0 *
	SD (±)	10.0	13.3	15.5	12.1	11.3
	95% CI	32.76–40.23	34.81–42.18	32.19–41.80	29.25–36.74	22.49–29.50
% sludge in gall bladder	GROUP 2	36.0	36.5	37.5	40.5	42.5
	SD (±)	11.7	10.8	9.5	11.4	9.5
	95% CI	32.75–39.24	33.50–39.49	34.86–40.13	37.34–43.65	39.86–45.13
	GROUP 3	35.0	30.5	27.5	24.5 *^,#^	20.0 *^,#^
	SD (±)	12.2	10.9	7.9	9.3	10.0
	95% CI	31.61–38.38	27.48–33.52	25.31–29.68	21.92–27.07	16.90–23.09
	GROUP 1	1.6	1.6	1.5	1.4	1.2
	SD (±)	0.5	0.5	0.5	0.5	0.4
	95% CI	1.44–1.75	1.44–1.75	1.34–1.65	1.24–1.55	1.07–1.32
Liver echogenicity	GROUP 2	1.6	1.6	1.6	1.4	1.3
	SD (±)	0.7	0.7	0.8	0.5	0.5
	95% CI	1.38–1.81	1.38–1.81	1.35–1.84	1.24–1.55	1.14–1.45
	GROUP 3	1.5	1.5	1.2	1.0 *	1.0 *
	SD (±)	0.7	0.5	0.4	0.0	0.0
	95% CI	1.28–1.71	1.34–1.65	1.07–1.32	1.00–1.00	1.00–1.00

CI (confidence interval). Exact *p*-values are reported in the text. * *p* < 0.05 compared with baseline (D0) value. ^#^ *p* < 0.05 compared among groups.

## Data Availability

All data generated or analysed during this study are included in this published article.
